# Intestinal Microbial Metabolites in Ankylosing Spondylitis

**DOI:** 10.3390/jcm10153354

**Published:** 2021-07-29

**Authors:** Giuseppe Scalise, Antonio Ciancio, Daniele Mauro, Francesco Ciccia

**Affiliations:** Rheumatology Unit, Department of Precision Medicine, Università Degli Studi Della Campania L. Vanvitelli, 80138 Naples, Italy; giuseppe.scalise@unicampania.it (G.S.); antonio.ciancio@unicampania.it (A.C.)

**Keywords:** ankylosing spondylitis, immunometabolism, microbial metabolism, microbiota, SCFA, dysbiosis, interleukin 17

## Abstract

Ankylosing spondylitis (AS) is a chronic inflammatory disease characterized by inflammation of axial joints and the pelvis. It is known that intestinal dysbiosis may exert direct pathogenic effects on gut homeostasis and may act as a triggering factor for the host innate immune system to activate and cause inflammation in extraintestinal sites in the so-called “gut-joint axis”, contributing to AS pathogenesis. However, although the intestinal microbiota’s influence on the clinical manifestation of AS is widely accepted, the mechanisms mediating the cross-talk between the intestinal lumen and the immune system are still not completely defined. Recent evidence suggests that the metabolism of microbial species may be a source of metabolites and small molecules participating in the complex network existing between bacteria and host cells. These findings may give inputs for further research of novel pharmacological targets and pave the way to applying dietary interventions to prevent the onset and ameliorate the clinical presentation of the disease. In this review, we discuss the role of some of the biological mediators of microbial origin, with a particular focus on short-chain fatty acids, tryptophan and vitamin B derivatives, and their role in barrier integrity and type 3 immunity in the context of AS.

## 1. Introduction

Immunometabolism has recently became an appealing area of translational research; metabolic cues regulate cell survival and differentiation and modulate the activity of a plethora of immune responses against microbes, cancer cells, and toward the self [1]. Microbial metabolism has been recognized as a source of small molecules influencing the behaviour of host immune cells contributing to the cross-talk between microbiota and host immune response [2]. In addition, recent data suggest the influence of the microbiota in determining the response to therapy in chronic arthritis [3]. However, the nature of the biological mediators involved in this interaction in physiological and pathological conditions is still not entirely known.

Emerging data are pointing beyond the known role of microbial-derived molecules such as lipopolysaccharide (LPS) and muramyl peptide in the activation of immune cells [4,5]. Bioproducts of microbial metabolism such as short-chain fatty acids, vitamin B, and tryptophan metabolites exerted immunomodulatory activity on the immune system’s different subtypes and non-immune cells, including intestinal epithelial cells [6].

Axial spondyloarthritis (AxSpA) is a chronic inflammatory disease belonging to spondylarthritis (SpA), characterized by inflammation of the axial joint [7]. Multiple studies demonstrate subclinical gut inflammation in up to 60% of patients affected by AxSpA [8,9]. Intestinal inflammation seems invariably linked to the dysfunction of the epithelial gut barrier, leading to a condition named “leaky gut” [8,10,11]. The inefficient separation of the intestinal lumen with the subepithelial space is associated with the aberrant activation of mucosal resident immune cells that, recirculating, may transfer the inflammation to the joint, establishing a pathogenic gut-joint axis [12,13,14,15]. The mucosal barrier alteration is part of a vicious circle involving the genetic predisposition, particularly HLA-B27, environmental factors, and altered intestinal dysbiosis [9,16]. Although the initial trigger of the dysbiosis is still not fully explained, multiple animal and human studies demonstrated an active role for the microbiota in the initiation and perpetuation of the inflammatory pathways behind the intestinal inflammation observed in AxSpA and in SpA more in general [8,17,18]. The study of the bacterial metabolic pathways and their bioproducts could offer another key to interpreting the maladaptive cross-talk existing between intestinal epithelial cells and mucosa immune cells and their immunometabolism which could be deeply helpful in deciphering the etiopathogenesis of these conditions.

This work reviews the main metabolomic studies that identified quantitative and qualitative alterations in metabolic alteration in AS, principally focusing on microbial-derived metabolites. In particular, the biological activity of microbial bioproducts will be summarized in the context of AxSpA pathogenesis, discussing their role in linking intestinal epithelial barrier inflammation and arthritis. Finally, the future therapeutic perspective harnessing microbial metabolic activity will be summarized.

### Methodology for Review of Published Literature

The work was written as a narrative review. Authors selected the most relevant works according to their judgment and then discussed them collegially. The electronic bibliographic databases Pubmed (National Library of Medicine, Bethesda, MD, USA, https://pubmed.ncbi.nlm.nih.gov/, accessed on 26 July 2021) and Google Scholar (Mountain View, CA, USA, https://scholar.google.com/, accessed on 26 July 2021) were interrogated in January 2021. To ensure that information collected was complete, the request was also performed on grey literature. The request combined words and expressions for conceptual groups including ”Metabolomics” and “Metabolites”, “Lipidomics”, “Short-chain fatty acid”, “Tryptophane”, “Vitamin B”; “Spondyloarthritis”, “Ankylosing Spondilitis”, “Arthritis”; “Gut”, “Intestine”, “Intestinal”, and “Microbiota”. Only papers published in English language were considered, and duplicate publications were deleted.

## 2. Metabolomics in AS: Overview

**Figure 1:**
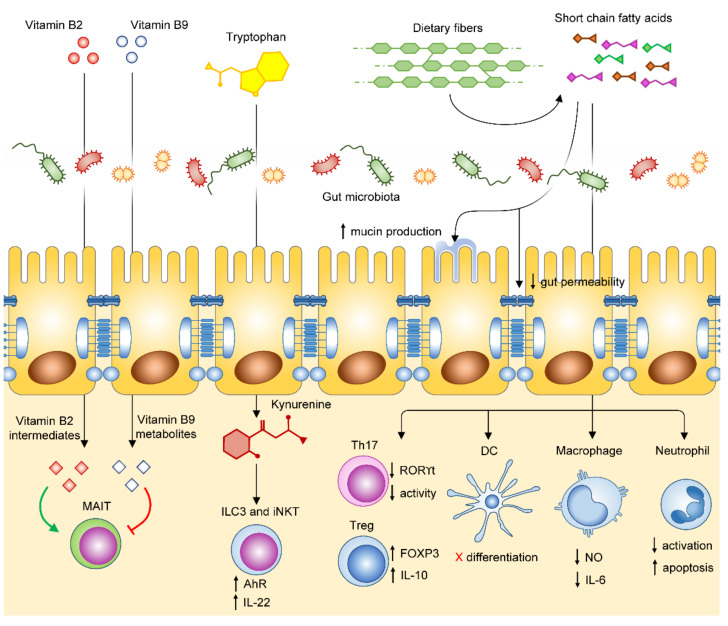
Schematic view of the effects of microbial metabolites on intestinal and immune cells. From left to right: vitamin B2 and B9 metabolites respectively activate and inhibit MAIT cells function; tryptophane metabolite kynurenine activates aryl hydrocarbon receptor (AhR) on iNKT and ILC3 cells and induces IL-22 production; SCFAs produced from microbic metabolism of dietary fibers interact with intestinal epithelial cells and promote mucine production and decrease paracellular permeability; absorbed SCFAs promote FoxP3 and IL-10 expression in regulatory T-cells (Tregs) and decrease RORϒt expression in Th17 lymphocytes, as well as Th17 activity; SCFAs block DC differentiation, decrease production of nitric oxide (NO) and IL-6 in macrophages, and inhibit neutrophil function and promote their apoptosis.

Metabolomics is a robust and comprehensive approach to characterize and evaluate small (<1–1.5 kDa) metabolic products in complex biological environments in a systematic fashion [19]. Metabolomics can provide information about the pathogenesis and, eventually, the activity of several rheumatic diseases since the alteration of metabolites concentrations can be associated with the activation of specific inflammatory pathways [20]. However, the availability of sophisticated metabolic profiling of AxSpA paired with the significant amount of data coming from other “omics” sciences, including transcriptomics and genomics, is soon offering clues in the understanding of the pathogenesis of autoimmune and inflammatory diseases. The key studies assessing metabolomics in AS patients are briefly summarized in Table 1 [21,22,23,24,25]. These studies pointed to three principal metabolic pathways here discussed: fat metabolism, amino acids metabolism, and intestinal microbial metabolism. In light of the evidence existing on the role of dysbiosis in SpA and the emerging knowledge on the immunobiology of SpA, the latter seems one of the most appealing fields and is discussed in more detail and summarized in Figure 1.

## 3. Microbial Metabolites in AS

Alterations of microbial composition have been demonstrated in patients with SpA, and those alterations may contribute to the shaping of intestinal immune response which in some cases is mirrored by the increased recirculation of immune cells expressing markers of intestinal homing [27,28]. More recently, Klingberg et al. analyzed the cecal microbiota composition in 150 patients with AxSpA, 18 patients with ulcerative colitis (UC), and 17 healthy controls (HC) with GA-map™ Dysbiosis Test, which also indicates the degree of deviation of the microbiota composition compared with healthy control populations. Dysbiosis was found in 87% of AxSpA patients [29].

The data on the perturbed microbial composition in SpA may be enriched from a functional perspective considering the metabolic studies performed in AxSpA that highlighted qualitative and quantitative alterations in chemical species derived from the microbial metabolism. In particular, metabolic alterations possibly originating from intestinal dysbiosis were found in two studies.

In the first one, Wang et al., using both plasma, urine, and ligament tissue from patients with AxSpA, found that urinary levels of glycine and hippurate were increased in AxSpA patients compared to healthy controls, while phenyl acetyl glycine (PAG) and butyrate were decreased [24]. Hippurate is a carboxylic acid commonly found in urine, produced by intestinal bacteria using glycine and benzoic acid, and increased urinary levels have been associated with gut dysbiosis [30]. Additionally, a decreased PAG level in the urine has also been associated with intestinal microbiota disorders [24]. In the second study, Shao et al. demonstrate the decrease in short-chain fatty acids (SCFAs) butyrate and propionate in axSpA patients’ fecal samples [25]. SCFAs are organic acids, mainly produced within the intestinal lumen by bacterial fermentation of undigested dietary carbohydrates [31]. Altered intestinal microbiota, and the subsequent perturbations of these metabolites, mainly SCFA, appear to have a crucial role in immunoregulation, mainly affecting the permeability of intestinal lining and the generation of tolerogenic T cells in the intestinal lumen. In a predisposed subject, these events may lead to the onset of the SpA [27].

In particular, HLA-B27 may have a predisposing role, and this has been demonstrated in an HLA-B27 transgenic mouse model. Asquit et al. performed metabolomic profiling of cecal content in this rat model before and after onset of the disease, and found a number of metabolites significantly upregulated versus controls, suggesting that metabolic changes may be an early event in AxSpA pathogenesis [32]. Amongst these, the authors found perturbations in spermidine, which is involved in tight junction formation and mucosal defense [33], or in histidine, tyrosine, and N-acetylmuramate levels that may reflect a switch towards differential usage of amino acid pathways that led to colonic protein fermentation [32].

### 3.1. Short-Chain Fatty Acids

The intestinal microenvironment is characterized by a finely tuned equilibrium between the host immune system and gut microbiota, where intestinal homeostasis is the result of the active interplay between them. Any action that may cause an unbalance towards either element, such as a locally overreactive immune system or intestinal dysbiosis, may result in disease. In the healthy gut, several bacterial species are responsible for SCFA production, with members of the *Firmicutes* phylum being the most important in the human colon for what concerns butyrate [34]. Altered gut microbiota may lead to a decreased production of SCFA and susceptibility to disease, as it seems in IBD. Studies from fecal samples in patients with UC and CD showed a reduction in butyrate producing species, and decreased fecal SCFA levels [35]. In SpA, perturbations in the gut microbial population’s composition are widely recognized, therefore suggesting the idea that dysbiosis may have a key role in the disease [27]. In a seminal study by Costello et al., ileal biopsies from recent-onset AxSpA patients who were naïve to anti-TNF treatment showed an increase in five microbial populations compared to healthy controls, namely *Lachnospiraceae*, *Ruminococcaceae*, *Rikenellaceae*, *Porphyromonadaceae*, *and Bacteroidaceae* and a decrease in the abundance of *Veillonellaceae* and *Prevotellaceae* [28]. However, while it is not yet known whether these alterations have repercussions on SCFA production in human, data from a study in transgenic HLA-B27/β2m rats, exhibiting alterations in intestinal microbiota similar to human counterpart [18], reported a moderate increase in SCFA levels [32].

After being locally produced by colonic microbiota, SCFAs become a readily available energy source for colonocytes. An early work estimated that butyrate oxidation accounts for more than 70% of the oxygen consumed by human colonocytes from the ascending and descending colon [36]. Besides their role as a readily available energy source for eukaryotic cells, SCFAs have been demonstrated to be critical mediators of intestinal homeostasis. Their overall effect seems to be protective towards the integrity of the intestinal environment, enhancing barrier function, as well as showing anti-inflammatory properties through interaction with several mediators of innate and acquired immunity [37].

SCFAs exert their effects mainly through two different pathways: interaction with G-protein coupled receptors (GPRs), GPR41 (FFAR3), GPR43 (FFAR2), and GPR109a (HM74b), and histone deacetylase (HDAC) inhibition. Each SCFA exhibits a different affinity for each GPR, with propionate having a higher affinity for GPR41 and GPR43 compared to the other two [38] and GPR109a having a selective affinity for butyrate and (D)-beta-hydroxybutyrate, apart from being a receptor for niacin [39]. After interaction with their ligand, the receptors activate Gi/G0 proteins with subsequent inhibition of adenylate cyclase [38,40]; however, GPR43 is also capable of coupling with Gq proteins [38], thus activating the phospholipase C-dependent pathway [41]. The three receptors are expressed in intestinal epithelial cells, as well as other cell types. Namely, GPR41 can be found in peripheral blood mononuclear cells (PBMC), dendritic cells (DC), polymorphonuclear leukocytes (PMN), spleen, lymph nodes, bone marrow, lung, and adipose tissue [38]; GPR43 is expressed in PMN, PBMC, monocytes, and lymphocytes as well [38]; GPR109a is present in macrophages, monocytes, PMN, DC, Langerhans cells and adipocytes [42]. Concerning HDAC inhibition, butyrate shows the highest activity among the other SCFAs [43]. HDAC are key enzymes that promote gene expression by rendering genome segments more accessible to transcription, and their inhibition accounts for many of the antiproliferative and anti-inflammatory properties of butyrate [44].

Butyrate is also known to activate peroxisome proliferator-activated receptors (PPAR)-γ, the ubiquitously expressed nuclear transcription factor involved in fatty acid storage and glucose metabolism [45,46,47]. Among its various effects, PPAR-γ activation also inhibits the NF-κB pathway, thus decreasing inflammation [48]. The relationship between PPAR-γ and inflammation has notable implications in inflammatory bowel diseases’ pathogenesis (IBD). Patients with UC show decreased PPAR-γ expression [49], and medications that act as PPAR-γ agonists, such as rosiglitazone and mesalazine, ameliorate intestinal inflammation [50,51]. Furthermore, PPAR-γ activation in intestinal epithelial cells by bacteria-derived butyrate is an important driver of energy expenditure towards β-oxidation of fatty acids and butyrate itself: this metabolic pathway, being more oxygen-consuming, is a major contributor to the physiological intestinal epithelial hypoxia [52]. A low oxygen partial pressure in the intestinal lumen is crucial in the healthy gut and shapes the intestinal microbiota in favor of SCFA-producing obligate anaerobes while inhibiting dysbiosis caused by Enterobacteriaceae [47].

The plethora of biological roles of SCFA can be divided into direct effects on intestinal lining and effects on inflammation, bone metabolism, and immune cells.

#### 3.1.1. Effects on the Intestinal Lining

SCFAs have been shown to enhance Mucin 2 (MUC2) expression in vitro in human goblet-like LS174T cells through HDAC inhibition and consequent histone acetylation and methylation at the level of *MUC2* promoter region, as well as via AP-1 activation [53]. This effect was, however, lost after incubation of goblet cells with higher doses of butyrate (>1 mM) and propionate (>15 mM). Jung et al. further demonstrated that incubation of LS174T cells with butyrate solution increased expression of MUC3, MUC4, and MUC12 with no effect on MUC2 expression. Mucin expression affects also bacterial adherence, promoting adhesion to cultured cells of *Lactobacillus acidophilus* and *Bifidobacterium longum* and inhibiting adhesion of *Escherichia coli* [54]. Interestingly, dysbiosis is promoted by adherent and invasive bacterial species such as *Escherichia coli* and *Prevotella* spp. characterizes ileal samples of patients with AxSpA [8]. However, mucin production, particularly MUC1, seems to be increased in the terminal ileum of AxSpA patients, along with an increased expression of IL-22 [55]. IL-22 has a protective role towards gut barrier integrity and immunity, and its expression appears to be promoted by butyrate in vitro and in vivo [56]. Moreover, butyrate supplementation in mice promotes IL-22 expression by CD4^+^ T cells and innate lymphoid cells (ILCs) and partially protects wild-type (WT) mice from *C. rodentium*-induced colitis. Conversely, *IL-22^−/−^* mice infected with *C. rodentium* experience more severe colitis than WT mice and show no benefit from butyrate supplementation, thus suggesting a crucial role for IL-22 as a critical mediator acting downstream butyrate signaling pathway [56].

Besides mucins, intestinal epithelial cells also produce a set of peptides with direct antimicrobial activity such as lectins, defensins, and cathelicidins, which have a fundamental role in regulating the local microbial population preventing bacterial overgrowth and dysbiosis.

Defensins are mainly produced by Paneth cells (PCs) at the bottom of the intestinal crypts and play an important role in the host defense against microbes. In AS patients with subclinical gut inflammation, PCs function is altered, leading to an overexpression of PC-related peptides, especially human α-defensin 5 (HD-5) [57].

Concerning cathelicidins, in vitro studies showed that SCFAs and particularly butyrate enhance cathelicidin LL-37 expression in human colonic cells. Interaction of SCFAs with GPR43 receptor promoted expression of type-C lectin RegIIIγ and β-defensins 1, 3, and 4 in cultures of murine and human intestinal epithelial cells [58,59].

Butyrate is an important promoter of intestinal barrier function through the upregulation of tight junction proteins. In vitro studies in Caco-2 cell monolayers revealed that butyrate facilitates Ca^2+^ dependent assembly of ZO-1 and occludin on the cellular periphery through AMPK activation [60]. Weaned piglets fed with a sodium butyrate diet experienced less pronounced diarrheal symptoms and a reduced intestinal permeability as evaluated through lactulose-mannitol ratio in urine; furthermore, butyrate fed piglets exhibited increased expression of ZO-1, occluding and claudin-3 in ileal samples and of claudin-3 alone in colonic samples. In the same study, Caco-2 cells were treated with sodium butyrate and showed decreased permeability and increased expression of ZO-1, occludin, and claudin-3. This effect was impaired by the treatment of Caco-2 cells with GPR109a-interfering shRNA [61]. Claudin-1 expression was also found to be promoted by butyrate in cdx2-IEC cells37, whereas the expression of claudin-2, a paracellular channel-forming protein that increases epithelial permeability, appears to be repressed under butyrate administration through an IL-10 Receptor-dependent pathway in Caco-2 and T84 cells [62].

Furthermore, a recent work evidenced the role in gut permeability of synaptopodin, a protein described initially for its crucial role in forming renal podocyte foot processes and postsynaptic densities of neuronal synapses. Butyrate administration on T84 cultured cells generated upregulation of the SYNPO gene and promoted tight junction assembly and monolayers formation. SYNPO knockdown cells could not form a proper monolayer and showed increased permeability and delayed wound healing, which was not adequately compensated by butyrate. In addition, SYNPO expression resulted in being hampered in T84 cells treated with TNF-α, IL-1β, and IFN-γ, suggesting that inflammation downregulates synaptopodin production. These findings were paralleled by observations in Synpo^−/−^ mice subjected to DSS-induced colitis: compared to control WT, knockout mice experienced more severe colitis, increased gut permeability, and did not exhibit any amelioration after butyrate treatment [37].

These data taken together may have a biological relevance if linked to some evidence coming from human patients affected by AxSpA. The connections between SCFAs and gut permeability may have notable implications in the pathogenesis and therapy of SpA. We have previously shown that gene expression analysis on ileal samples from AS patients exhibit a decreased expression of intestinal tight junction proteins, such as claudin-1, claudin-4, occludin and zonula occludens protein 1, and of endothelial tight junction proteins, causing permeabilization of the gut vascular barrier, translocation of bacterial products in the bloodstream, and subsequent inflammation. This effect was accompanied by a significant upregulation of zonulin, the mammalian equivalent of zonula occludens toxin produced by *Vibrio cholerae*, which antagonizes the formation of tight junctions at the epithelial and endothelial level [8].

Zonulin appears also to be a potential therapeutic target: in collagen-induced arthritis (CIA) mouse, a mouse model of inflammatory arthritis characterized by an impaired intestinal permeability and gut microbial dysbiosis, dietary supplementation with butyrate reduced serum zonulin concentration and upregulated the expression of tight junctions protein, thus restoring gut permeability. Interestingly, zonulin targeting (both indirectly with butyrate and directly with larazotide acetate) prevented arthritis onset and attenuated arthritis symptoms, supporting the hypothesis of the gut-joint axis [10].

#### 3.1.2. Effects on Inflammasome, Neutrophils, Macrophages, and DCs

SCFAs are known to have direct anti-inflammatory effects. Inhibition of NF-kB transcription factor is a common ending point that SCFAs achieve through different pathways, such as HDAC inhibition and PPAR-gamma activation [45,48,63]. NF-kB downregulation leads to a decreased expression of several actors of the inflammatory response, such as pro-inflammatory cytokines, chemokines, iNOS and COX-2 enzymes, adhesion molecules, growth factors, acute phase proteins, and immune receptors [64].

Interestingly, SCFAs proved to be capable of suppressing LPS-induced autophagy and NLRP3 inflammasome activation in Caco-2 cells. The two effects appeared to be reliant on two different mechanisms, the former being seemingly dependent on SCFAs role as energy substrates and the latter being a consequence of HDAC inhibition. Both autophagy and NLRP3 inflammasome activation showed deleterious effects on intestinal barrier integrity, and any of the two systems can mutually activate the other [65]. Autophagy is a physiological mechanism in which part of cytosol regions, comprising organelles, are sequestered in vesicular compartments and undergo controlled digestion. This process is needed to replace damaged intracellular machinery and provide a way of rapidly recycling nutrients while coping with starvation and infections in the gut microenvironment [66]. In ileum samples of AxSpA patients with chronic gut inflammation, autophagy is significantly upregulated in the intestinal epithelium and lamina propria mononuclear cells (LPMC) and is accompanied by an increased expression of IL-23p19 by LPMC [67]. NLRP3 inflammasome activation has a relevant role in AxSpA patients as evidenced in a study from our group, which found significant overexpression of NLRP3 in the inflamed gut of HLA-B27 rats, SKG mice, and human AS patients [68]. Treating SKG mice with MCC950 (an NLRP3 antagonist) before induction of the inflammatory disease with curdlan suppressed gut disease development and delayed the onset of articular inflammation [68]. Moreover, activation of NLRP3 inflammasome appeared to be correlated with gut dysbiosis since antibiotic treatment in HLA-B27 rats reduced NLRP3 expression, whereas, in human patients, the expression levels of NLRP3, NLRC4, and AIM2 were positively correlated with the number of adherents and invading bacteria found in ileal samples from AxSpA patients [68]. Interestingly, isolated bacteria from ileal samples of AxSpA patients, but not from healthy controls, induced NLRP3 overexpression in PBMC from healthy controls [68].

In mice, neutrophils treated with SCFAs are more sensitive to apoptosis, have decreased killing activity [69] and decreased chemotaxis [70]; these effects are seemingly mediated by SCFA-GPR43 interaction. Surprisingly, however, GPR43 deficient mice appear to suffer from more severe acute colitis but are protected from chronic colitis [71]. Chang et al. demonstrated that butyrate has an immunomodulatory effect on macrophages. Murine bone marrow-derived and intestinal lamina propria macrophages were stimulated with LPS and either butyrate, acetate, or propionate. They found that levels of nitric oxide, IL-6, and IL-12p40 were strongly decreased in the presence of butyrate in a dose-dependent manner, and this effect was attained through HDAC inhibition [72].

SCFAs regulate DCs function at multiple levels. Singh et al. demonstrated that propionate and butyrate block differentiation of DCs from bone marrow progenitors in mice. After entrance inside dendritic progenitor cells through transporter SCL5A8, SCFAs acted as inhibitors of HDAC1 and HDAC3 isoforms of HDAC, with consequent repression of PU.1 and RelB, two transcription factors that are crucial in the differentiation of DCs [73]. On the other hand, SCFAs appear to target CD103^+^ DCs, which have intestinal homing and contribute to intestinal T differentiation and induce tolerogenic effects in the intestinal mucosa by promoting differentiation of FoxP3^+^ Treg cells [74].

A high-fiber diet and direct butyrate and acetate administration in mice promoted the tolerogenic activity of CD103^+^ DCs in mesenteric lymph nodes through increased expression of retinaldehyde dehydrogenase-2 (RALDH2). This enzyme catalyzes the conversion reaction from vitamin A to retinoic acid, ultimately promoting regulatory T cell (Tregs) differentiation [75]. This effect resulted in being GPR43- and GPR109a- mediated [75]. In addition, GPR43-dependent response to acetate promotes IgA production by mucosal B-cells through CD103^+^ DCs and retinoic acid production [76].

It is worth mentioning that in murine models of experimental autoimmune uveitis, propionate treatment ameliorated eye inflammation [77]. In the same study, Nakamura et al. used Kaede/C57BL/6J transgenic photoconvertible mice to investigate potential trafficking of T cells from the gut to the eye. Kaede mice are engineered to ubiquitously express the photoconvertible protein Kaede, which irreversibly changes its fluorescence from green to red spectrum upon photoactivation with near-UV light. By applying photoconversion in the colonic lumen and later searching for red fluorescence in the eye, it was possible to prove the trafficking of leukocytes from the gut to the inflamed eye [77]. Propionate treatment in these mice decreased trafficking of Th1 cells from the gut to extraintestinal sites and led to a diminished number of T cells in the inflamed eye, although no statistical comparison could be performed on pooled eyes given the small number of effector T-cells found in the eyes of the propionate-treated mice [77]. This effect could be a consequence of propionate treatment on chemokines’ expression, such as CCL2 [77].

#### 3.1.3. Effects on Bone Metabolism

In AxSpA, bone metabolism is altered dually since there is a coexistence of both increased bone resorption and new bone juxtaposition [78]. One of the goals of current research is to tackle both processes to prevent spinal fusion and reduced mobility in AS patients. SCFAs seem to provide an overall positive effect on bone composition in experimental models of arthritis. Using three independent experimental approaches, namely direct supplementation of SCFA, high-fiber diet (HFD), and bacterial transfer, Lucas et al. found that mice fed with direct SCFA supplementation and HFD had increase systemic bone density, reduced bone resorption, and reduced osteoclasts [79]. They found that the ability of SCFA to suppress osteoclast differentiation and bone resorption was independent of receptors GPR41 and GPR43, but both butyrate and propionate induced a metabolic switch in osteoclasts towards glycolysis and significantly suppressed TRAF6, an essential osteoclastogenic signaling component [80]. Therefore, the authors addressed the role of SCFA and HFD on inflammatory bone loss in two experimental arthritis models: CIA mouse model and serum-induced mouse (SIA) model. SCFA treatment significantly attenuated the severity of inflammation, systemic bone mass was increased after treatment, and osteoclast-specific gene expression in the bones was significantly downregulated [79]. Therefore, in light of the perturbation of bone homeostasis featuring AxSpA, further studies are warranted to dissect the contribution of intestinal derived SCFAs to the aberrant bone remodeling associated with SpA.

#### 3.1.4. Metabolic Regulation of T Cells Subsets

Human studies in SpA and animal models demonstrated T cells’ participation in the pathogenesis of both articular and extraarticular manifestations of AS [13,81].

Notably, among T cells, aberrant activation of Th1 and Th17 is a plausible culprit in the disease manifestation. Similarly, Tregs, specialized in terminating the host immune response, are significantly increased in the inflammation site, including synovial fluid and ileum of SpA compared to the bloodstream. This increase could be considered an attempt to counteract the inflammation associated to SpA [82]. Conversely, in HLA-B27/β2m transgenic rat altered ratio between IL-17/IL-10 has been observed, which partially depends on a defective IL-10 production from Tregs [13,83]. In the same animal models, SCFAs seems to be paradoxically increased rather than decreased, suggesting a possible anti-inflammatory response.

Interestingly, the downregulation of the SCFA receptor GPCR43 may suggest a resistance toward the SCFA action in this model [32,84]. SCFA can epigenetically control the differentiation and function of the immune cells. In particular, it has been demonstrated that SCFAs regulate T cell plasticity.

In a work from Chen et al., naïve CD4^+^ T cells from CBir1 transgenic mice (a mouse model of inflammatory colitis) were cultured in either Th1 or Th17 conditions and then treated with butyrate. Under Th1 conditions, the butyrate-treated cells produced IL-10 and IFN-gamma, whereas, in Th17 promoting conditions, butyrate inhibited IL-17 production and promoted IL-10 production. Transfer of these butyrate-treated cells in Rag1^−/−^ mice, in which both B and T cell development is impaired, induced less severe colitis than non-butyrate-treated cells [85]. The transcription factor Blimp-1 seems to be a crucial mediator for butyrate-dependent production of IL-10 by Th1. However, there is contrasting evidence about whether this mechanism is mediated by GPR43 interaction or HDAC inhibition [86]. SCFAs, particularly butyrate, also have a role in CD8^+^ T cells activation, as they induce IFN-gamma upregulation and increased production of effector molecules such as granzyme B [87].

Moreover, SCFA, mainly butyrate and propionate, can induce Treg differentiation by regulating FOXP3 expression. Smith et al. demonstrated that germ-free mice had reduced colonic concentrations of SCFAs and lower numbers of colonic T regulatory cells (cTregs) [88]. SCFA administration in these mice was associated with an expansion of the pool of Foxp3^+^ IL-10 producing cTregs by increasing de novo generation of inducible Treg cells in the colonic lamina propria. This effect is mediated by HDAC inhibition in a GPR43-dependent manner. They also found that this receptor is overexpressed in intestinal Tregs compared to Tregs isolated from spleen or mesenteric lymph nodes. These results have also been confirmed in knockout mice for GPR43 [88]. The effect of SCFAs on Tregs generation has been further investigated in a mouse model of colitis; Zhang et al. showed that butyrate treatment ameliorated TNBS-induced colitis in mice and this effect was sustained by an increased number of peripheral blood CD25^+^ Foxp3^+^ Tregs, with subsequent higher levels of peripheral blood and colonic IL-10 and IL-12, and a decreased level of IL-17 and IL-23 in mesenteric lymph nodes [89]. In vitro studies on human PBMCs showed that butyrate promotes CD25^+^ Foxp3^+^ Tregs differentiation in a dose-dependent manner [89] and reduces Th17 differentiation, and leads to a decreased production of pro-inflammatory cytokines, including IL-23, in LPS-activated PBMCs [90].

The role of SCFA on T cell plasticity has also been evaluated in the HLA-B27/β2m transgenic rat. Asquith et al. demonstrated that the oral administration of propionate to HLA-B27/β2m rats significantly reduced both cecal and colonic intestinal inflammation assessed histologically and reduced mRNA expression of the inflammatory mediators IL-1β, IFNγ and, interestingly, IL-17A. Therefore, they determined the frequency of CD4+ FoxP3+ T cells in the cecum, colon, spleen, and mesenteric lymph nodes following SCFA administration, but surprisingly, they did not observe a higher Treg frequency [32].

The effect of SCFAs on Th17 differentiation was further explored by Sałkowska et al.; by culturing naive CD4^+^ cells with butyrate and apcidin (an HDAC inhibitor), they showed a significant reduction of RORγt (retinoic acid receptor-related orphan receptor-γt; essential transcription factor for Th17 generation), IL17A and IL17F. Surprisingly, however, differentiated Th17 cells and Jurkat T cells treated with butyrate and apcidin showed enhanced RORγt expression [91].

One possible interpretation of these data is that either quantitative/qualitative alteration in SCFA content or an impaired signaling downstream GPR43 in patients with AS may contribute to an impaired Treg function. This alteration could be behind the unbalanced type 3 immune response, resulting in the activation of IL23-IL17/22 axis and of the mucosal cell involved in type 3 immunity and regulated by RORγt such as ILC3, invariant natural killer T (iNKT) cells, mucosal-associated invariant T (MAIT) cells, and γδ-T cells [14].

### 3.2. Vitamin B Metabolites

Group B vitamins are water-soluble, and unlike lipid-soluble vitamins, they cannot be stored by the body, thus requiring appropriate dietary intake in order to avoid deficiencies. Commensal bacteria may also provide a source of B vitamins, and their interaction with the intestinal environment has direct effects on intestinal immune cells. As previously discussed, niacin (vitamin B3) is a GPR109A agonist like butyrate and promotes gut barrier function and Treg differentiation and exerts anti-inflammatory effects by modulating pro-inflammatory cytokine secretion [42,61,92]. GPR109A activation by niacin may modulate microbiota-induced intestinal inflammation mediated by ILC3. Bhatt et al. showed that Gpr109a^−/−^Rag1^−/−^ mice, which are incapable of producing mature T and B lymphocytes and a proper adaptive immunity while also lacking GPR109A, spontaneously developed colitis whereas classic Rag1^−/−^ mice housed in the same cage did not [93]. Colonic samples of Gpr109a^−/−^Rag1^−/−^ mice showed an increased number of ILC3 and an increased production of IL-17 compared to Rag1^−/−^ mice [93].

Interestingly, colonic DCs from Gpr109a^−/−^Rag1^−/−^ mice were able to promote ILC3 expansion in vitro significantly more than DCs from Rag1^−/−^ mice and this effect was inhibited by IL-23 blockade [93]. Finally, both Gpr109a^−/−^Rag1^−/−^ and Rag1^−/−^ mice were administered niacin in the drinking water, but only Rag1^−/−^ mice showed decreased ILC3 in colonic samples after treatment [93]. Taken all together, these data suggest that niacin-GPR109A interaction inhibits IL-23 production by colonic DCs and consequently hampers ILC3 development in colonic mucosa. ILC3 are a class of lymphocytes capable of prompt immune responses that comprises lymphoid tissue inducer cells (LTi), NKp44^+^ and NKp44^−^ cells [11]. While LTi cells are required for the development of Peyer patches in the colon, NKp44^+^ and NKp44^−^ ILC3 are involved in protection against luminal pathogens and are the innate equivalent of Th22 and Th17 lymphocytes of the adaptive immunity [11]. In AxSpA patients, ILC3 have been demonstrated to be expanded in the gut, peripheral blood, synovial fluid, and bone marrow [94]. Interestingly, a high percentage of ILC3 in the bone marrow showed increased expression of integrin α4β7, a homing marker that binds integrin receptor MAdCAM1, and the latter appears to be significantly overexpressed in the gut and bone marrow of AS patients compared to controls [94]. These data suggest that there may be an aberrant recirculation of ILC3s from the gut to the bone marrow, where they act in close proximity to the sites of joint inflammation [94]. Therefore, in keeping with ILC3 primary mucosal origin, microbial-derived vitamin B3 may act as the biological meditator of ILC3 activation, possibly in SpA.

Vitamin B2 (riboflavin) is found in milk, eggs, liver, and leafy green vegetables. As evaluated in a metagenomic analysis by Magnusdottir et al., potential riboflavin bacterial producers are Bacteroidetes and Fusobacteria and most Proteobacteria and half of the Firmicutes [95]. An intriguing characteristic of riboflavin intermediate metabolites is their ability to promote activation of mucosal associated invariant T cells (MAIT cells) [96]. These cells are a distinct population of T cells that exhibit predominant innate-like behaviors and, unlike classic T lymphocytes, express a restricted TCRα and β chains repertoire [97]. Therefore, MAIT cells can be activated by signaling pathways different from TCR; MHC-related protein 1 (MR1), a non-polymorphic major histocompatibility complex (MHC) class I-like molecule expressed on antigen-presenting cells [97], confers an innate like-behavior to MAIT cells. MR1 protein can bind small molecule antigens, such as vitamin B2 and B9 metabolites that can be produced by bacteria [96]. Riboflavin metabolites appear to elicit MR1-dependent activation of MAIT cells, while 6-formyl pterin, a vitamin B9 intermediate, does not appear to have any stimulating effect [98]. This microbiota-induced activation of MAIT cells may have notable implications in the pathogenesis of AS [13]. It is worth noting that, in humans, the primary localization of MAIT cells are the mucosal surfaces, but they are particularly abundant also in the liver and in circulation, where they represent 45% and 10% of total T cells, respectively, and represent one of the most abundant sources of IL-17 [99]. IL-17A^+^ MAIT cells are expanded in synovial fluid of AS patient, and in addition to IL-17, they are able to produce IL-22, confirming their relevance in AS where they correlate with the disease activity score [100,101].

In contrast to riboflavin, vitamin B9 (folate) possesses direct immunomodulatory effects [102,103]. Dietary sources of folate are leafy green vegetables, beans, and liver and among commensal bacteria, nearly all Bacteroidetes, and most Fusobacteria and Proteobacteria, possess folate biosynthesis pathway [95]. Treg cells express high levels of vitamin B9 receptor FR4 (Folate receptor 4) [104] and vitamin B9 may act as a survival factor for Treg [102]. Cultures of CD4^+^ T cells in B9-depleted conditions were associated with a reduced number of Tregs and a decreased expression of anti-apoptotic factor Bcl-2, without any effect on the differentiation of Tregs from naïve T cells [102]. In addition, mice fed with a folate-deficient diet showed a decreased frequency of colonic Tregs and an increased susceptibility to TNBS-induced colitis [103].

In summary, microbial-derived vitamin B metabolites seem to exert a dual effect, stimulatory and immunomodulatory, on MAIT cells. Therefore, the intricate balance between the diet and the different microbial species and their metabolisms may result in both a pro-inflammatory and anti-inflammatory phenotype of intestinal MAIT cells in AS.

### 3.3. The Role of Tryptophan and Its Metabolites

Regarding amino acid metabolism, the results from different metabolomic studies indicate inconsistent amino acid perturbations. This discrepancy may be explained by the small sample size of each study and the different approaches used (mass spectrometry versus NMR spectroscopy). Nevertheless, it is worth noticing that two main amino acid alterations were confirmed in different “omics” studies (reported in Table 1): glutamine and tryptophan (Trp). In particular, Gao et al. found a decreased level of Trp in plasma of AxSpA patients compared to healthy controls [22], suggesting an increased metabolization of the Trp by the host cells, while Berlinberg et al., using intestinal tissue samples and matched fecal samples metagenomics studies, detected significant alterations in Trp pathway metabolites sustained by bacteria metabolism [26].

In the human intestine, the Trp metabolism occurs principally in the intestinal epithelial cells, immune cells, and in enterochromaffin cells also entering in the microbial metabolism [105,106,107,108]. Trp deprivation activates the ribosomal kinase GCN2 triggering a stress response and promote autophagy [109]. Whether the reduced Trp levels observed in SpA may be linked to the upregulation of autophagy associated with AS is still unknown [67]. Consistently, Trp rich diets ameliorate the dextran sodium sulfate-induced colitis in mice and piglets [107,110]. The Trp effect on human immune response seems, however, mainly mediated by Trp metabolism.

Host metabolism of Trp relies mainly on the enzyme indoleamine 2,3 dioxygenase (IDO1), which is induced by the inflammatory milieu and by dysbiosis [106,111]. Its localization is mainly inside the intestinal immune cells and intestinal epithelial cells, accounting for 95% of Trp metabolism [112]. IDO1 is a potent immunoregulatory enzyme promoting immune actions on antigen-presenting cells and promoting Treg cells’ development and immune tolerance. The immunoregulatory properties of IDO1 are further supported by the evidence that IDO1^−/−^ mice are more prone to develop colitis-associated to increase pro-inflammatory cytokine secretion and Treg suppression in the colon [113]. The enzymatic activity of IDO1 on Trp leads to kynurenine production, which can modulate immune cells and epithelial function through the activation of aryl hydrocarbon receptor (AhR) and indirectly shapes the intestinal microbiota [112,114,115].

Recently, AhR upregulation has been detected in circulating iNKT cells isolated from AxSpA patients and in ILC3 within the peripheral SpA synovial tissue [116,117]. In both cases, AhR seems to be associated with IL-22 rather than with IL-17 production. This may suggest anti-inflammatory feedback in the light of the protective role of IL-22 on intestinal barrier integrity and the expansion of IL-22 producing ILC3 observed in AS [94,118].

Moreover, 4–6% of total Trp can be directly metabolized by gut bacteria, through the action of the enzyme tryptophanase, into tryptamine and indolic byproducts [26,105]. Some of these indole metabolites, such as IPA (indole propionic acid) and IAA (indole acetic acid), act as a ligand for the AhR and pregnane X receptor (PXR), regulating intestinal barrier function [107,119,120]. *CARD9* is a susceptibility gene for inflammatory bowel disease, and it has been shown that CARD9 promotes recovery from colitis through the production of IL-22. *Card9^−/−^* mice appear to be more susceptible to colitis, have dysbiosis, and a reduction in colonic IL-22 expression. It has been postulated that the altered microbiota of *Card9^−/−^* mice were less capable of producing IAA and other AhR ligand necessary for the IL-22 production contributing to the hypersusceptibility to colitis [119]. Consistently, IAA levels were reduced in *Card9^−/−^* mice and the transfer of *Card9^−/−^* intestinal microbiota to germ-free wild type mice was sufficient to impair IL-22 activation and to increase the sensitivity to colitis [119]. Moreover, a recent study associating intestinal samples metabolomics with matched fecal samples metagenomic profiling, demonstrated how microbiota from ax-SpA patients switched from Trp synthesis to Trp metabolism, compared to both healthy controls and Crohn’s disease controls, suggesting that this alteration is specific to ax-SpA regardless of the presence of bowel inflammatory diseases [26].

Finally, a small portion of Trp (1–2%) can give rise to the serotonin production pathway in enterochromaffin cells via Trp hydroxylase 1 (TpH1), the expression of which is also regulated by SCFA. The intestinal-derived serotonin’s relevance in SpA is still under-investigated but may be relevant for the emerging role of serotonin in controlling bone remodeling and the existence of an entero-bone axis [121,122].

These and other evidence depict an intricate cross-regulation involving microbiota, diet, genetic, mucosal barrier, and immune function where it seems virtually impossible to delineate a hierarchy as each of these elements is reciprocally influenced. Genetic background, dysbiosis, and inflammation may shift this delicate balance promoting the increase in some Trp metabolites and reducing others, fueling or blunting the intestinal and systemic inflammation. Interestingly, metabolomic data from cecal content of HLA-B27/β2m transgenic rats showed a significant upregulation of the tryptophan derivatives kynurenine and N-acetylkurenine [32], and alteration in intestinal microbial tryptophan metabolism seems a feature of pediatric spondyloarthritis [123].

### 3.4. Trimethylamine and Trimethylamine-N-Oxide

Trimethylamine (TMA) is another molecule derived from the metabolic activity of intestinal microbiota on nutrients. Phosphatidylcholine, choline, and L-carnitine, which are contained in many foods, such as meat, fish, eggs, and dairy, are converted to TMA by intestinal bacteria; once absorbed TMA undergoes oxidation by flavin-containing monoxigenases (FMOs) in the liver, generating trimethylamine-N-oxide (TMAO) [124,125,126].

Metabolomic studies in human identified eight primary TMA producers among human microbiota, *Anaerococcus hydrongenalis*, *Clostridium asparagiforme*, *Clostridium hathewayi*, *Clostridium sporogenes*, *Edwardsiella tarda*, *Escherichia fergusonii*, *Proteus penneri*, and *Providencia rettgeri*, which are able to produce TMA from choline [127]. Besides phosphatidylcholine, choline, and L-carnitine, marine fish are a direct source of TMAO which does not require synthesis by intestinal microbiota [128].

While TMA and TMAO have been so far linked to increased cardiovascular risk by exerting pro-inflammatory and pro-atherogenic effects [126,129,130], there are also emerging data about a possible implication in spondyloarthritides. Although the implications of TMA and TMAO have not been studied yet in AS, is known from a recent work by Coras et al. that blood TMAO concentrations from patients with psoriatic arthritis positively correlated with joint and skin disease activity [131].

In a mouse model of graft-versus-host disease, TMAO diet supplementation has been shown to cause more severe disease and induce Th1 and Th17 lymphocyte differentiation and M1 macrophage polarization in an NLRP3 inflammasome-dependent way [132]. Intriguingly, NLRP3 inflammasome activation by TMAO in cultured mouse endothelial cell monolayers caused a decreased expression of ZO-1 proteins and disassembly of tight junctions, with subsequent increase of permeability [129].

Given the established role of Th17 lymphocytes and the potential implication of ZO-1 decreased expression in the pathogenesis of AS, these findings might provide another interesting link between intestinal microbiota and inflammation, whether these results would be replicated in experimental models of AS or AxSpA.

## 4. Conclusions and Future Directions

Significant progress has been made in the clinical and pathogenic understanding of AS over the past decade. Major advances in the role of innate immune cells and their cytokine signaling pathways have improved our understanding of this condition. Although the disease’s pathogenesis remains unclear, alterations of intestinal microbial composition have been demonstrated in patients with SpA and associated with intestinal and systemic immune alterations.

A complete understanding of the pathogenesis of AS, like most acquired diseases in humans, is needed in order to find mechanistically targeted therapies and achieve successful therapeutic responses. In this review, we showed how dysbiosis is associated with metabolic perturbations that lay down the onset of these inflammatory intestinal conditions, mainly through the downregulation of Treg suppressive signaling and the simultaneous upregulation of the IL23/17 axis. In light of this, immunometabolism can decipher the metabolic cues implicated in this dysregulation and how these perturbations can be modulated.

So far, attempts at modifying dysbiosis in AS patients through probiotics attained disappointing results [133], suggesting that the loss of gut barrier function and local inflammation may act only as a trigger, and once established, the inflammatory process proceeds independently. However, intervening in barrier integrity itself seems to achieve promising results in the management of joint inflammation. Some interesting perspectives are offered by research in rheumatoid arthritis, in which, in recent times, the role of gut dysbiosis and intestinal barrier integrity are being investigated [10]. In the CIA mice model, treatment with larazotide acetate, a recently developed zonulin receptor antagonist currently under evaluation for treatment in coeliac disease, led to the restoration of intestinal barrier integrity and decreased joint inflammation [10]. In the context of SpA-related animal models, the evidence that butyrate and propionate administration in transgenic HLA-B27/β2m rats attenuates bowel inflammation may give future perspectives for the use of prebiotics in the therapy of spondyloarthritides [32].

This strategy can create a new road in finding potential pharmacological targets to modulate the imbalance between Treg anti-inflammatory response and type 3 immune cells in the gut, blocking the gut-joint axis.

## Figures and Tables

**Table 1:** Main studies assessing metabolomics in AS patients.

	Patients	Approach	Biological Sample	Increased Metabolites	Decreased Metabolites
Zhou et al. [21]	30 AS, 32 RA, 30 HC	UPLC-TQ-MS	serum	leucine, valine, tryptophan, alanine, creatine, tyrosine, 4-hydroxy- L-proline, arginine, isoleucine, methionine, histidine, lysine	glutamine, glutamate, phenylalanine, serine, proline, γ-aminobutyric acid, creatinine, dimethyl-glycine, taurine, asparagine, acetyl- carnitine, ornithine, citrulline, threonine, glycine, aminobutyric acid
Gao et al. [22]	15 AS (all males), 24 HC	GC-MS and LC-MS	plasma	proline, glucose, phosphate, urea, glycerol, phenylalanine, homocysteine	phosphocholines, tryptophan, bipeptide phenylalanyl-phenylalanine
Gupta et al. [23]	81 SpA (33 axial, 47 peripheral), 86 HC	1H-NMR spectoscopy	serum	alanine, glutamate, glutamine, proline, phenylalanine, histidine, valine, leucine, isoleucine, acetate, choline, N-acetyl glycoproteins, N-α-acetyl-lysine, creatine/creatinine	acetone, LDL/VLDL, polyunsaturated lipids
Wang et al. [24]	PLASMA/URINE:44 AS, 44 HCLIGAMENT TISSUE:30 AS, 30 FNF	1H-NMR spectroscopy	plasma, urine, ligament tissue	PLASMA: 3-hydroxybutyrate, NAG, methionine, acetone, acetoacetate, betaine, glycerol URINE: glycine, hippurate, 2-pyridone-3-carboxamide (2-PY) LIG. TISSUE: triglycerides	PLASMA: leucine, valine, alanine, triglycerides, glucose, glutamate. URINE: butyrate, glutamate, creatinine, phenylacetyl-glycine (PAG) LIG. TISSUE: choline
Shao et al. [25]	40 AS, 35 RA, 34 HC	1H-NMR spectroscopy	feces	taurine, methanol, fumarate, tryptophan	butyrate, propionate, methionine, hypoxanthine
Berlinberg et al. [26]	24 HC, 27 CD, 21 axial SpA, 12 CD+axial SpA	LC-MS	Intestinal biopsies	indole-3-acetate (IAA), indole-3-acetaldehyde (I3Ald)	omega-3 fatty acid metabolites

AS: ankylosing spondylitis; RA: rheumatoid arthritis; CD: Crohn’s disease, HC: healthy control; SpA: spondyloarthritis; FNF: femoral neck fracture; UPLC-TQ-MS: ultra-high-performance liquid chromatography-triple quadrupole mass spectrometry; GC-MS: gas chromatography-mass spectrometry; LC-MS: liquid chromatography-mass spectrometry; NMR: nuclear magnetic resonance.
